# Comparative analysis of the genomes and aflatoxin production patterns of three species within the *Aspergillus* section *Flavi* reveals an undescribed chemotype and habitat-specific genetic traits

**DOI:** 10.1038/s42003-024-06738-w

**Published:** 2024-09-13

**Authors:** Alexandra Schamann, Sebastian T. Soukup, Rolf Geisen, Sabine Kulling, Markus Schmidt-Heydt

**Affiliations:** Department of Safety and Quality of Fruit and Vegetables, Max Rubner-Institut, Federal Research Institute of Nutrition and Food, Karlsruhe, Germany

**Keywords:** Fungal genomics, Food microbiology

## Abstract

Aflatoxins are the most dangerous mycotoxins for food safety. They are mainly produced by *Aspergillus flavus*, *A. parasiticus*, and *A. minisclerotigenes*. The latter, an understudied species, was the main culprit for outbreaks of fatal aflatoxicosis in Kenya in the past. To determine specific genetic characteristics of these *Aspergillus* species, their genomes are comparatively analyzed. Differences reflecting the typical habitat are reported, such as an increased number of carbohydrate-active enzymes, including enzymes for lignin degradation, in the genomes of *A. minisclerotigenes* and *A. parasiticus*. Further, variations within the aflatoxin gene clusters are described, which are related to different chemotypes of aflatoxin biosynthesis. These include a substitution within the *afl*L gene of the *A. parasiticus* isolate, which leads to the translation of a stop codon, thereby switching off the production of the group 1 aflatoxins B_1_ and G_1_. In addition, we demonstrate that the inability of the *A. minisclerotigenes* isolates to produce group G aflatoxins is associated with a 2.2 kb deletion within the *afl*F and *afl*U genes. These findings reveal a relatively high genetic homology among the three *Aspergillus* species investigated. However, they also demonstrate consequential genetic differences that have an important impact on risk-assessment and food safety.

## Abstract (web summary)

*Aspergillus minisclerotigenes*, linked to fatal aflatoxicosis in Kenya, shares high genetic similarity with *A. flavus* and *A. parasiticus*. However, genome analysis reveals key differences in aflatoxin gene clusters and enzymes, crucial for food safety and risk assessment.

## Introduction

*Aspergillus* subgenus *Circumdati* section *Flavi* comprises many species that are relevant for food and nutrition in contrastive ways. On the one hand, certain species, such as *Aspergillus oryzae*, are utilized in the production of fermented foods like koji and miso^1^. On the other hand, many representatives produce mycotoxins and thus pose a threat to food safety. At least 18 different species in the *Aspergillus* section *Flavi* synthesize aflatoxins, which have been classified as group 1 carcinogens^2,3^. Of these, the most important species are *A. flavus* and *A. parasiticus*, which have been repeatedly isolated from cereals and nuts^4–6^. *A. parasiticus* is described as preferring ground crop hosts such as peanuts, whereas *A. flavus* infects a wider range of hosts^7^. It is noted that isolates of *A. flavus* L strain, which are characterized by the formation of a few large sclerotia (>400 nm in diameter) but numerous conidia, are better adapted to the phyllosphere compared to isolates of *A. flavus* S strain^8,9^. In contrast, the latter ones form many small sclerotia (<400 nm) but only a few conidia, indicating that they are better adapted to the soil habitat^8,10^. *A. minisclerotigenes*, another highly aflatoxigenic *Aspergillus* species, was initially isolated from peanuts and has been consistently found in maize samples^11–13^. However, the preferred habitat of this species has not yet been defined. Knowledge about the preferred habitat and chemotype of fungal species allows for the assessment of health risks associated with a contaminated crop. The presumed chemotype and also the preferred habitat can be deduced to some extent from the interpretation of genomic data. For aflatoxigenic *Aspergillus* species, for example, different aflatoxin chemotypes exist due to modifications within the aflatoxigenic genes in the form of deletions, insertions, or single nucleotide polymorphisms, despite the structure of the aflatoxin gene cluster, which includes these approximately 30 genes necessary for the biosynthesis of aflatoxins, is mostly conserved^14,15^. *A. parasiticus* and *A. minisclerotigenes* have been described as capable of producing all the four main aflatoxins B_1_ (AFB_1_), B_2_ (AFB_2_), G_1_ (AFG_1_) and G_2_ (AFG_2_). In contrast, most aflatoxigenic *A. flavus* strains only produce AFB_1_ and AFB_2_^3,11,16^. This is due to different deletions in *A. flavus* genomes, including the 5’-end of the coding regions of the *afl*F and *afl*U genes, as well as their intergenic region, at the 5’-end of the aflatoxin gene cluster. These two genes are necessary for the production of G-group aflatoxins and code for a cytochrome P450 monooxygenase and an aryl alcohol dehydrogenase^17,18^. The evolutionary background behind the diversity of various aflatoxin chemotypes and the benefit for the fungus to produce either all or only certain aflatoxins remain unclear. Additionally, the preference for a specific habitat based on genomic data has not been determined until now. Aflatoxins are particularly problematic for food safety in regions with warm and humid climates, like sub-Saharan Africa. In Eastern Kenya, for example, high levels of contamination were detected in maize samples from households. In detail, 75% of the controlled samples contained aflatoxins at levels that exceeded the regulatory limit in Kenya (10 µg/kg)^19^. The consumption of contaminated food can result in chronic and acute toxic effects^20^. A significant outbreak of acute aflatoxicosis, resulting in 317 documented cases and 125 deaths, occurred in Eastern Kenya in 2004 due to the consumption of contaminated homegrown maize^21^. Highly aflatoxigenic *Aspergillus* sp. strains, which are genetically more closely related to *A. minisclerotigenes* than to *A. flavus* and are endemic to Eastern Kenya, were identified as the responsible strains for this outbreak of acute aflatoxicosis^22^. Later, it was stated that these “lethal aflatoxicosis fungus” strains fell within the genetic diversity of *A. minisclerotigenes*^23^.These strains are also characterized by the inability to form G-group aflatoxins due to a deletion of 2.2 kb within the aflatoxin gene cluster, which includes parts of the *afl*F and *afl*U genes^22^. To our knowledge, the whole genome sequence of such a strain has not yet been thoroughly investigated until now.

Some species in the *Aspergillus* section *Flavi* produce the mycotoxin cyclopiazonic acid (CPA) in addition to aflatoxins. CPA is an indole-tetramic acid that has been found to cause, among others, neurological disorders and gastrointestinal complaints in animal studies^24^. In *A. flavus*, there are three genes (*mao*A, *dma*T, and *pks-nrps*) that encode the proteins for CPA biosynthesis. Additionally, there is one gene (*ctf*R1) that appears to regulate the expression of these structural genes. These genes are located in a small gene cluster near the 5’-end of the aflatoxin gene cluster^25^. *A. parasiticus* is described as being unable to produce CPA^3,26^ and the inability to amplify genes of the CPA gene cluster suggests a deletion in this species^26^. However, the exact extent of this modification has not yet been described. In addition to the aforementioned knowledge gaps, no comprehensive analysis has been conducted at the genomic and analytical levels for important *Aspergillus* section *Flavi* species found in Eastern Kenya. However, this analysis is essential in the event of an aflatoxicosis outbreak to quickly predict the severity and extent of the disease and make an appropriate risk assessment in the case of maize infestation with aflatoxin-producing fungi. Thus, in this study, the genomes of strains of *A. flavus*, *A. minisclerotigenes*, and *A. parasiticus* were compared to each other. For this, genomic data of the strains *A. flavus* MRI19, *A. minisclerotigenes* MRI390 and MRI400, and *A. parasiticus* MRI410, which were recently sequenced by us^27–29^, was used and analyzed in detail. Partial gene sequences of calmodulin, nitrate reductase, and *ß*-tubulin revealed a high homology between strain *A. minisclerotigenes* MRI390 and strains of the so-called lethal aflatoxicosis fungi (e.g., *Aspergillus* sp. A1168)^28^. Indications for the preferred habitat of these different species were investigated at the genomic level. Further, secondary metabolite biosynthetic gene clusters (BGCs) were analyzed with a specific focus on the aflatoxin gene cluster. These clusters were associated with the mycotoxin patterns (chemotypes) produced by the respective strains.

## Results

### Genome annotation

Characteristics of the genome assemblies of *A. flavus* MRI19, *A. minisclerotigenes* MRI390 and MRI400, and *A. parasiticus* MRI410 are published elsewhere^27–29^. The ab initio eukaryotic gene finding of the genome assemblies using Augustus (v. 3.4.0) predicted the highest number of protein coding genes in the genome of *A. parasiticus* (11,914). This was followed by *A. minisclerotigenes* MRI390 (11,551) and MRI400 (11,501), as well as *A. flavus* (11,489), which were mostly annotated (Table 1).

**Table 1:** Characteristics of the genome assemblies and annotation of *A. flavus* MRI19, *A. minisclerotigenes* MRI390 and MRI400, and *A. parasiticus* MRI410

Parameters	*A. flavus* MRI19	*A. minisclerotigenes* MRI390	*A. minisclerotigenes* MRI400	*A. parasiticus* MRI410
No. of predicted genes	11,489	11,551	11,501	11,914
No. of annotated genes (Gene Ontology)	9841	9754	9871	10,109
Total no. of BUSCO orthologs	1706	1706	1706	1638
Complete single-copy, complete multicopy, fragmented, and missing orthologs (%)	93.7, 0.9, 0.5, 4.9	93.7, 0.9, 0.5, 4.9	93.4, 0.8, 0.6, 5.2	96.0, 0.9, 0.1, 3.0

### Whole-genome alignment

**Fig. 1:**
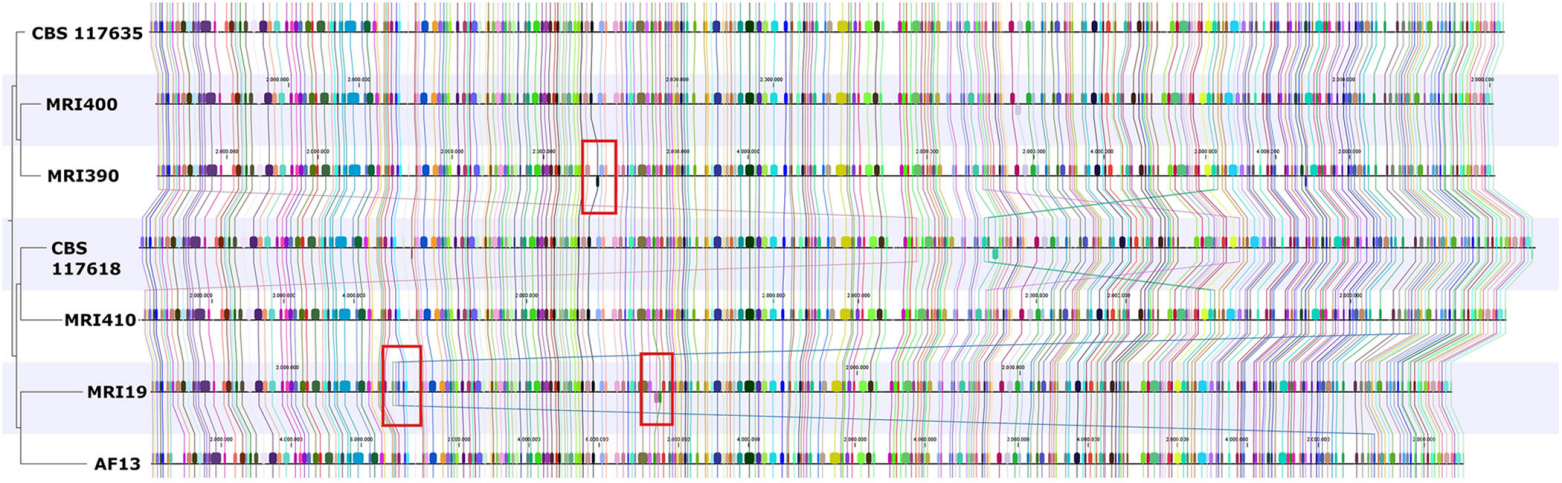
Whole-genome sequence-based synteny analysis. A whole-genome alignment was performed using the CLC Genomics Workbench for the genome assemblies of *A. flavus* MRI19, *A. minisclerotigenes* MRI390 and MRI400, and *A. parasiticus* MRI410. Additionally, the genome assemblies of *A. flavus* AF13 (GenBank ACCN: GCA_014117485.1)^30^, *A. minisclerotigenes* CBS 117635 (ACCN: GCA_009176455.1)^31^, and *A. parasiticus* CBS 117618 (ACCN: GCA_009176385.1)^31^ were included for comparison. Modifications mentioned in the text are marked in red.

A whole-genome alignment was performed on the four sequenced strains and in addition, for comparison, with *A. flavus* AF13^30^, *A. minisclerotigenes* CBS 117635^31^, and *A. parasiticus* CBS 117618^31^. A high overall similarity with some larger modifications among the different species was revealed (Fig. 1). In the genome of *A. flavus* MRI19, two variations were detected in relation to the aforementioned strains. An approximately 360 kb fragment present on chromosome 8 of the other strains was aligned to chromosome 2 in *A. flavus* MRI19. Further, on chromosome 4, an approximately 210 kb fragment was invertedly incorporated. In *A. minisclerotigenes* MRI390, approximately 223 kb of chromosome 2 were observed to be inverted compared to the other strains. In addition, smaller modifications were detected. Based on the alignment, an average nucleotide identity of more than 93% was calculated across the whole genomes between all strains (Table 2). The average nucleotide identity between the *A. flavus* and *A. minisclerotigenes* isolates was approximately 97.0%, between *A. flavus* and *A. parasiticus* approximately 93.9%, and between *A. minisclerotigenes* and *A. parasiticus* approximately 94.0%.

**Table 2:** Average nucleotide identity [%] (above the diagonal) and alignment percentage [%] (below the diagonal) between *A. flavus*, *A. minisclerotigenes*, and *A. parasiticus* genomes

Fungal strains	*A. flavus* MRI19	*A. flavus* AF13	*A. minisclerotigenes* MRI390	*A. minisclerotigenes* MRI400	*A. minisclerotigenes* CBS 117635	*A. parasiticus* MRI410	*A. parasiticus* CBS 117618
*A. flavus* MRI19		99.46	97.00	96.98	97.00	93.85	93.88
*A. flavus* AF13	96.65		96.97	96.95	96.98	93.83	93.86
*A. minisclerotigenes* MRI390	90.80	89.92		99.16	98.88	93.97	93.98
*A. minisclerotigenes* MRI400	91.22	90.30	96.53		98.88	93.97	93.98
*A. minisclerotigenes* CBS 117635	92.29	91.23	94.95	95.41		93.96	93.97
*A. parasiticus* MRI410	87.60	86.73	86.86	87.29	87.99		98.57
*A. parasiticus* CBS 117618	88.08	87.09	87.01	87.45	88.45	94.92	

### Strain-specific genes

**Fig. 2:**
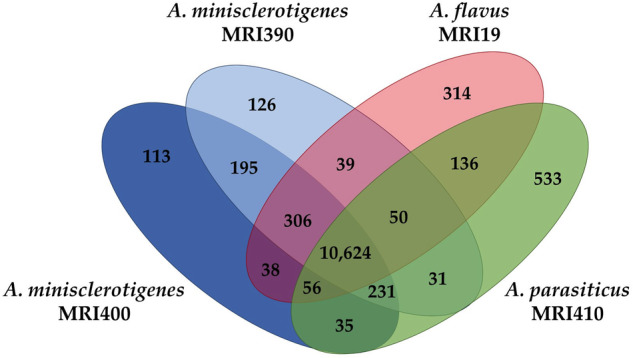
Venn diagram illustrating the numbers (mean values) of homologous and strain-specific genes identified in the genomes of *A. flavus* MRI19, *A. minisclerotigenes* MRI390 and MRI400, and *A. parasiticus* MRI410. Genes were classified as strain-specific if they had <50% coverage and/or <50% similarity to the nucleotide sequences of all genes in the other strains.

Strain-specific genes were identified among the predicted genes in the genomes of *A. flavus* MRI19, *A. minisclerotigenes* MRI390 and MRI400, and *A. parasiticus* MRI410. These genes were defined as having <50% similarity or coverage to the nucleotide sequences of genes from the other sequenced strains. Based on these criteria, more than 10,600 genes were identified as homologous (Fig. 2). The genome of *A. parasiticus* MRI410 had the highest number of strain-specific genes (533, Fig. 2). An enrichment analysis was conducted to compare the strain-specific genes of an isolate with all predicted genes of the same isolate. The analysis revealed that *A. flavus* MRI19 is most over-represented in the Gene Ontology (GO) terms “endopeptidase activity” (GO:0004175) and “serine-type endopeptidase activity” (GO:0004252). The genome of *A. parasiticus* MRI410 is most over-represented in the GO terms “carbon-oxygen lyase activity, acting on phosphates” (GO:00016838) and “pyruvate dehydrogenase activity” (GO:0004738). The most enriched GO terms for the strain-specific genes of *A. minisclerotigenes* were “anchored component of membrane” (GO:0031225) and “cell adhesion mediator activity” (GO:0098631) for strain MRI390, and “DNA integration” (GO:0015074) and “corticosterone binding” (GO:1903875) for strain MRI400 (Supplementary Data 1).

### Carbohydrate-active enzymes (CAZymes)

**Fig. 3:**
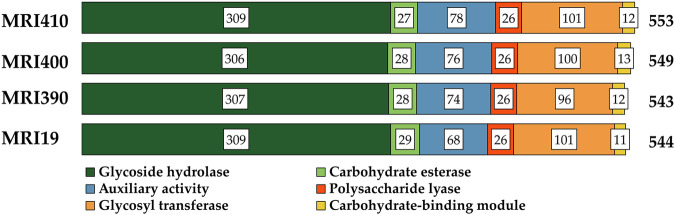
Carbohydrate-active enzymes (CAZymes) were identified in the genomes of *A. flavus* MRI19, *A. minisclerotigenes* MRI390 and MRI400, and *A. parasiticus* MRI410 using the dbCAN2 meta server. These enzymes were classified into different enzyme modules.

CAZymes, which are enzymes required for the synthesis, modification, and degradation of complex carbohydrates and glycoconjugates, serve as indicators of a fungal species’ ability to metabolize specific carbon sources^32,33^. CAZymes are also relevant for the degradation of host cell wall components, making them crucial in infection and colonization processes. CAZymes were identified in the genomes of *A. flavus* MRI19, *A. minisclerotigenes* MRI390 and MRI400, and *A. parasiticus* MRI410 and were classified into the different modules based on their function, including carbohydrate esterase, auxiliary activity (AA), glycoside hydrolase (GH), glycosyl transferase, polysaccharide lyase, and carbohydrate-binding module^32^. Overall, a high similarity in the identified families and subfamilies was observed among the sequenced strains (Fig. 3, Supplementary Data 2). The highest deviation was detected for module AA, which includes catalytic enzymes involved in the degradation of plant cell walls. A total of 78 enzymes were detected for *A. parasiticus* MRI410, 74 and 76 for *A. minisclerotigenes* MRI390 and MRI400, but only 68 for *A. flavus* MRI19. Here, the most striking differences were seen in the family AA4 and the subfamily AA3_2, which include lignin-degrading and oxidizing enzymes^34^. Among these enzymes, the lowest number was found in *A. flavus* MRI19 (AA4: 0, AA3_2: 24). However, more enzymes were detected in *A. minisclerotigenes* (MRI390: AA4: 1, AA3_2: 27; MRI400: AA4: 2, AA3_2: 29) and *A. parasiticus* MRI410 (AA4: 3, AA3_2: 28) (Supplementary Data 2). Further differences include the increased presence of *α*-rhamnosidases from the family GH78 in the GH module, but a decreased availability of chitinases from the family GH18 and *β*-1,3-glucanases from the family GH55 in *A. flavus* and *A. parasiticus* compared to both strains of *A. minisclerotigenes* (Supplementary Data 2).

### BGCs

**Fig. 4:**
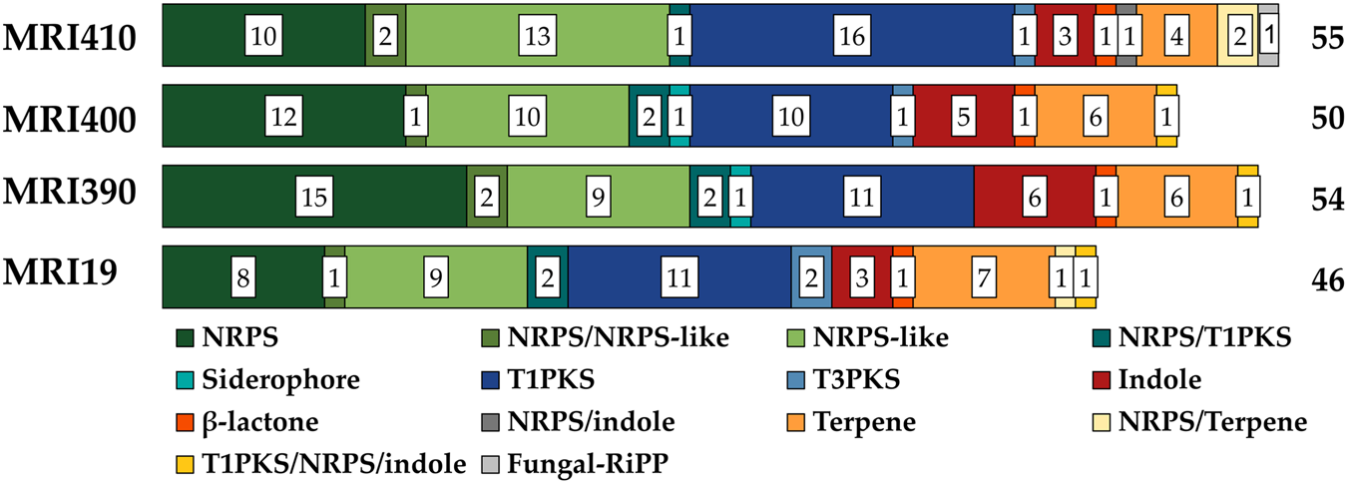
Secondary metabolite biosynthetic gene clusters were predicted in the genomes of *A. flavus* MRI19, *A. minisclerotigenes* MRI390 and MRI400, and *A. parasiticus* MRI410 using the fungal version of antiSMASH. Gene clusters were categorized into different groups based on their backbone gene(s) (NRPS, non-ribosomal peptide synthetase; T1PKS, type I polyketide synthase; T3PKS, type III polyketide synthase; RiPP, post-translationally modified peptide).

BGCs were identified in the genomes of *A. flavus* MRI19, *A. minisclerotigenes* MRI390 and MRI400, and *A. parasiticus* MRI410 using the fungal version of antiSMASH and were categorized into the following groups based on the key enzyme of the gene cluster: non-ribosomal peptide synthetase (NRPS) clusters, NRPS-like clusters, siderophore clusters, type I polyketide synthase (T1PKS) clusters, type III polyketide synthase (T3PKS) clusters, indole clusters, *β*-lactone containing protease inhibitor clusters, fungal post-translationally modified peptide (RiPP) clusters, and terpene clusters. Additionally, hybrid clusters have been detected. Differences could be observed among the sequenced *Aspergillus* species (Fig. 4). The genome of *A. parasiticus* MRI410 had the highest number of BGCs (55), followed by *A. minisclerotigenes* MRI390 (54). In the genomes of *A. minisclerotigenes* strains MRI390 and MRI400, one siderophore cluster was detected on contigs aligned to sequences of the database of the National Center for Biotechnology Information assigned to chromosome 3. The nucleotide sequence was mostly deleted in the other genomes. Within the - compared to the other strains - invertedly incorporated fragment on chromosome 4 of *A. flavus* MRI19 (see section “Whole-genome alignment”), an unknown BGC with a NRPS as the backbone gene was identified. Further, only in the genome of *A. parasiticus* MRI410 a RiPP cluster was detected.

### Aflatoxin gene cluster

**Fig. 5:**
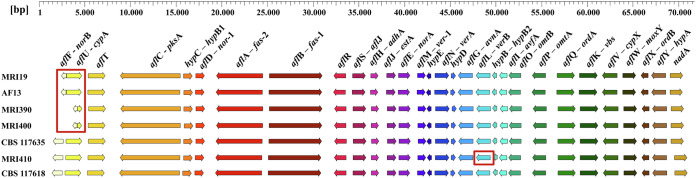
Comparison of the aflatoxin gene cluster among *Aspergillus* species. Genes of the aflatoxin gene cluster from *A. flavus* MRI19, *A. minisclerotigenes* MRI390 and MRI400, and *A. parasiticus* MRI410 were visualized using GenVision Pro. For comparison, the gene sequences of the following strains were used from the database of the NCBI: *A. flavus* AF13 (GenBank ACCN: GCA_014117485.1)^30^, *A. minisclerotigenes* CBS 117635 (ACCN: GCA_009176455.1)^31^, and *A. parasiticus* CBS 117618 (ACCN: GCA_009176385.1)^31^. Modifications mentioned in the text are marked in red.

The aflatoxin gene cluster, which was identified by antiSMASH as a T1PKS/NRPS/indole hybrid cluster in *A. flavus* MRI19 and *A. minisclerotigenes* MRI390 and MRI400, and as a T1PKS cluster in *A. parasiticus* MRI410, was found in all the four analyzed genomes on a contig that was assigned to chromosome 3. Among the four strains, a nucleotide identity of > 94% for the complete clusters and >96% for only the coding sequences (*A. flavus*/*A. minisclerotigenes* > 96.6%, *A. flavus*/*A. parasiticus* > 96.6%, *A. parasiticus*/*A. minisclerotigenes* > 96.2%, *A. minisclerotigenes* MRI390/*A*. *minisclerotigenes* MRI400 > 98.4%) was calculated using the CLC Genomics Workbench. Further, the gene clusters were visualized using GenVision Pro (Lasergene v. 17, DNASTAR, Inc, Madison, USA) (Fig. 5). All known genes of the aflatoxin gene cluster were present in all the seven analyzed strains, including *A. flavus* AF13^30^, *A. parasiticus* CBS 117618^31^, and *A. minisclerotigenes* CBS 117635^31^ in addition to the aforementioned four strains. The comparison of the aflatoxin gene cluster among the different *Aspergillus* species revealed that the majority of variations were observed at the 5’-end within the genes *afl*F and *afl*U. A deletion of 2176 bp, which includes the 5’-end of both genes and the entire intergenic region, was detected in the genomes of *A. minisclerotigenes* MRI390 and MRI400 compared to *A. minisclerotigenes* CBS 117635. Further, in *A. flavus* MRI19, a fragment of 939 bp including also the 5’-end of both genes and the complete intergenic region was deleted.

A further deletion of 23 bp was observed in the transcribed region of *afl*R in *A. minisclerotigenes* MRI390 and MRI400, but not in *A. minisclerotigenes* CBS 117635 or the other fungal species. All additional detected extensive variations were located in intergenic regions, such as a 401 bp deletion in the intergenic region between *afl*Y and *nad*A in all *A. flavus* and *A. minisclerotigenes* strains, in comparison to both *A*. *parasiticus* strains. As a further small but highly relevant modification, a substitution (G → T) was detected within the coding sequence of the gene *afl*L of *A. parasiticus* MRI410 at the 430th base of this gene. This led to the translation of the stop codon TAG, suggesting that this gene is non-functional. The gene *afl*L encodes the desaturase that converts versicolorin B to versicolorin A and thus introduces the double bond in the furan ring for the synthesis of group 1 aflatoxins^15,35,36^. Thus, this step is the branching point for the formation of group 1 and 2 aflatoxins.

### CPA gene cluster

**Fig. 6:**
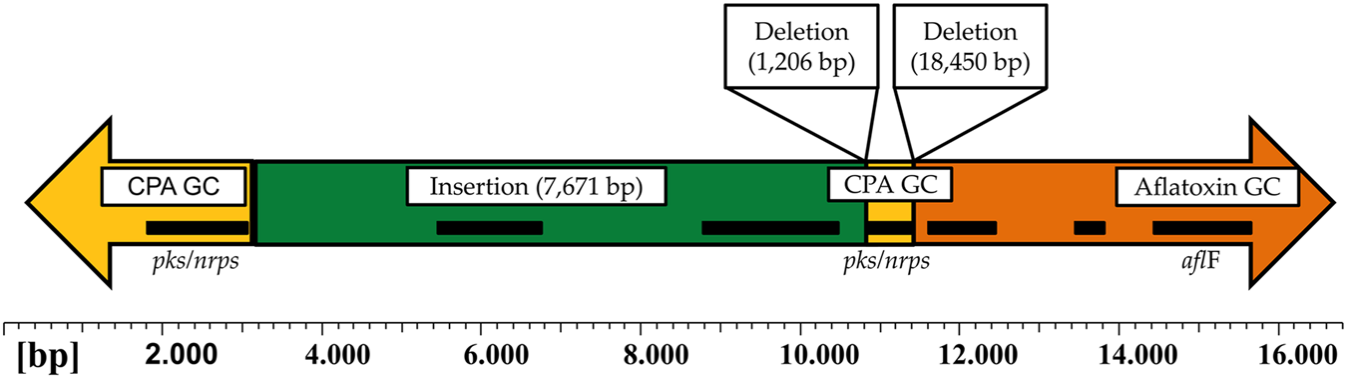
Schematic presentation of the region between the cyclopiazonic acid (CPA) gene cluster (GC) and the aflatoxin gene cluster of *A. parasiticus* MRI410. The lengths of the insertion and the deletions were determined by aligning the region that includes the remnants of the CPA gene cluster and the 5’-end of the aflatoxin gene cluster of *A. parasiticus* MRI410 with the corresponding sequence of *A. minisclerotigenes* MRI390. The genes located within this region are indicated by black bars.

The complete CPA gene cluster or its remnants were identified in the genomes of *A. flavus* MRI19, *A. minisclerotigenes* MRI390 and MRI400, and *A. parasiticus* MRI410 located near the 5’-end of the aflatoxin gene cluster, and thus also on contigs assigned to chromosome 3. The alignment of this region revealed a high homology between *A. flavus* MRI19 and *A. minisclerotigenes* MRI390 and MRI400. These strains contained the complete gene cluster. In the genome of *A. parasiticus* MRI410, the genes *dma*T and *mao*A were completely deleted as most of its *pks*/*nrps* gene (Fig. 6). A deletion of 18,450 bp starting approximately 3200 bp away from the 5’-end of the *afl*F gene of the aflatoxin gene cluster was detected, spanning over most of the CPA gene cluster. Between two remaining fragments of the *pks*/*nrps* gene, a second deletion of 1206 bp and an insertion of 7671 bp were detected in the genome of *A. parasiticus* MRI410. The inserted sequence contained two genes encoding a putative gluconolactone oxidase and a glucose oxidase precursor. This sequence was also found in the genomes of *A. minisclerotigenes* and *A. flavus*. In both *A. minisclerotigenes* strains, it was, however, located close to the end of the contig representing the 3’-end of chromosome 3. In *A. flavus* MRI19, it was located approximately 6620 bp away from the 5’-end of chromosome 3. In the genome of A. *parasiticus* MRI410, no additional copies of the inserted sequence were detected elsewhere.

### Quantitation of aflatoxins

**Fig. 7:**
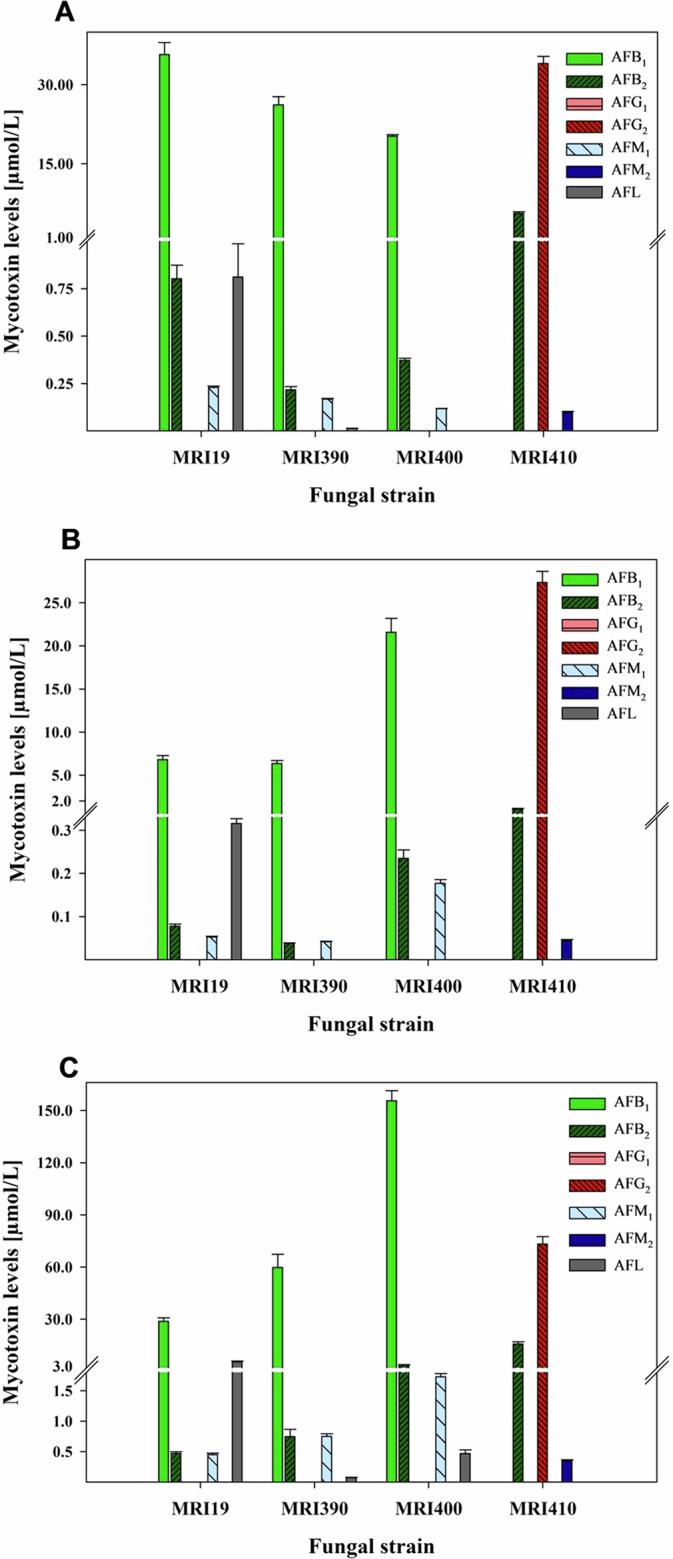
Aflatoxin levels formed by *Aspergillus flavus* MRI19, *A. minisclerotigenes* MRI390 and MRI400, and *A. parasiticus* MRI410 on MEA, CYA, and YES medium. Aflatoxin levels were measured by LC-MS in extracts of *A. flavus* MRI19, *A. minisclerotigenes* MRI390 and MRI400, and *A. parasiticus* MRI410 after 7 days of incubation on MEA (**A**), CYA (**B**), and YES (**C**), respectively. The values are mean values respectively ± standard deviation. Limit of quantitation for all analytes: 0.01 µmol/L. AFB_1_, aflatoxin B_1_; AFB_2_, aflatoxin B_2_; AFG_1_, aflatoxin G_1_; AFG_2_, aflatoxin G_2_; AFM_1_, aflatoxin M_1_; AFM_2_, aflatoxin M_2_; AFL, aflatoxicol.

To determine if the different aflatoxin chemotypes correspond to the genetic-structural variations observed in the aflatoxin gene clusters, the fungal strains were incubated for 7 days on malt extract agar (MEA), Czapek yeast autolysate agar (CYA), and yeast extract sucrose agar (YES), and their biosynthesized aflatoxins were measured. Growth rates on laboratory media were similar among the different species, with all reaching a colony diameter of 7.3–7.7 cm on MEA, 7.9–8.8 cm on CYA, and 8.7–8.9 cm on YES. Aflatoxin levels in methanolic extracts were measured using LC-MS with high mass resolution. For the identification of the aflatoxins in the samples, the accurate masses, retention times, isotope ratios, and MS/MS spectra were compared to those of the corresponding standards. Formed aflatoxin levels are shown in Fig. 7 and in the Supplementary Data 3 and 4. The levels varied strongly between extracts of the same stain incubated on different media and between different strains incubated on the same medium. AFB_1_ was the most abundant aflatoxin produced by *A. flavus* MRI19 and *A. minisclerotigenes* MRI390 and MRI400, regardless of the medium on which the three strains were incubated. *A. flavus* produced the highest amounts of AFB_1_ on MEA, whereas the *A. minisclerotigenes* strains produced the highest amounts on YES (Supplementary Data 3 and 4). Except for incubation on MEA, *A. minisclerotigenes* MRI400 formed clearly higher levels of AFB_1_ than *A. flavus* MRI19 and *A. minisclerotigenes* MRI390. AFB_2_ and aflatoxin M_1_ (AFM_1_) were produced on the three media in low levels of approximately 1–2% compared to AFB_1_, and even lower levels of aflatoxin M_2_ (AFM_2_) but no AFG_1_ and AFG_2_ were detected in extracts of the three strains. This fits to their deletion that was observed within the *afl*F and *afl*U genes. Further, aflatoxicol (AFL), a hydroxylation product of AFB_1_, was analyzed. Among the investigated strains, the highest levels of AFL were measured in extracts of *A. flavus* MRI19, regardless of the medium on which they were incubated. Within the three individual strains (*A. flavus* MRI19, *A. minisclerotigenes* MRI390 and MRI400), the highest levels of AFL were detected in extracts of fungi incubated on YES.

**Fig. 8:**
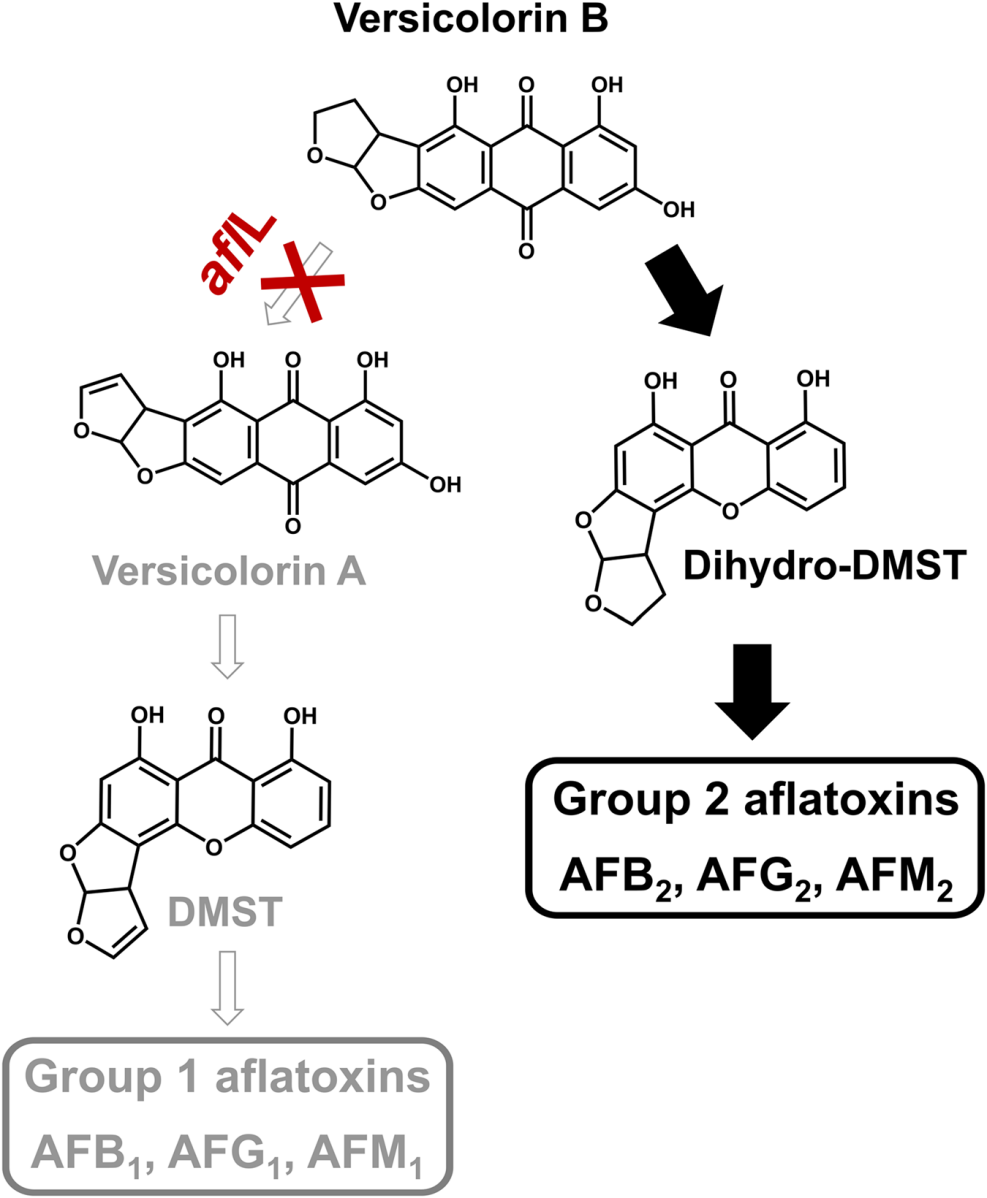
Schematic presentation of a part of the aflatoxin (AF) biosynthesis pathway of *A. parasiticus* MRI410. Due to a substitution within the *afl*L gene, which encodes the desaturase responsible for converting versicolorin B to versicolorin A, versicolorin B is mostly converted into group 2 aflatoxins. The known structures are based on Caceres et al.^15^. DMST, demethylsterigmatocystin.

*A. parasiticus* MRI410 showed a significantly different chemotype compared to the other strains (Figs. 7 and 8). The aflatoxin produced in the highest quantity by this strain was AFG_2_, which was measured at the highest levels in extracts of the fungus incubated on YES medium. AFB_2_ was biosynthesized by *A. parasiticus* MRI410 in relative amounts of 3.87% on CYA, 16.38% on MEA, and 21.45% on YES in relation to AFG_2_ levels. Further, AFM_2_ was measured at relatively low levels in all extracts, regardless of the medium on which *A. parasiticus* MRI410 was incubated. Low amounts of AFB_1_ and AFG_1_ could be detected at concentrations below the limit of quantitation, but not AFM_1_ or AFL. Additionally, CPA was detected in extracts of *A. flavus* and *A. minisclerotigenes*, but not in those of *A. parasiticus*. However, as CPA was not the focus of this publication, the LC-MS method was not optimized and validated for this mycotoxin and CPA was thus only semi-quantified.

### Identification of precursors of aflatoxins

The identification of aflatoxin precursors was not the main focus of this publication. However, since full mass scans were generated in high-resolution of the samples, it was also possible to search selected precursors of aflatoxins in extracts of *A. parasiticus* MRI410 without quantifying these compounds. The identification was based on the accurate masses and isotope patterns, as well as a comparison of the MS/MS spectra with respective data in the literature, if available^37,38^. No precursors of group 1 aflatoxins, which are typically formed in the biosynthetic pathway after the desaturation of versicolorin B to versicolorin A by AflL, such as sterigmatocystin, O-methylsterigmatocystin, and 11-hydroxy-O-methylsterigmatocystin, were detected. In contrast, versicolorin B and other precursors of group 2 aflatoxins, such as dihydro-O-methylsterigmatocystin and dihydro-11-hydroxy-O-methylsterigmatocystin, were detected (Supplementary Data 5). Additionally, a metabolite with a mass of *m/z* 329.0665 and the suggested formula C_17_H_12_O_7_ was detected by LC-MS in methanolic extracts of *A. parasiticus* MRI410, but not in those of the other investigated strains. Based on the evaluation of the respective MS/MS spectrum and the accurate mass, it could be suggested that this analyte may be a metabolite of versicolorin B. The compound differs from versicolorin B (C_18_H_12_O_7_) in accurate mass by 12.0003 Da and in molecular formula most likely by one fewer carbon atom.

## Discussion

The discrepancy between the high structural conservation of the aflatoxin gene cluster observed in the species *Aspergillus* on the one hand and the diversity reflected in the different chemotypes on the other hand, i.e., the evolutionary background of which chemotype generates which benefit for the producing fungus in its specific habitat, can be determined by analyzing the genome. The aim of the study was therefore to comprehensively compare the genomes of several toxicologically relevant strains of *Aspergillus* section *Flavi* at both the genetic and analytical levels. These strains have different profiles of aflatoxin biosynthesis. In the genome of the analyzed *A. parasiticus* strain, a substitution was detected within the *afl*L gene of the aflatoxin gene cluster. This gene codes for a desaturase, which disables this strain from producing significant levels of group 1 aflatoxins (AFB_1_, AFG_1_, AFM_1_). Further, both analyzed *A. minisclerotigenes* strains contain a deletion that includes the 5’-end of the coding sequences of *afl*F and *afl*U in the aflatoxin gene cluster. This deletion prevents the biosynthesis of G-group aflatoxins. In addition, this study has provided clear references of genetic indicators for the adaption of species to certain habitats. However, it has to be considered that only one respectively two representative strains of each species were included in the study.

*A. parasiticus* has been described as preferring ground crop hosts and is mostly isolated from soil or peanut samples^7^. In contrast, L strain isolates of *A. flavus* appear to prefer the phyllosphere^10,39^. The preferred habitat of *A. minisclerotigenes* has not been described yet. Appropriately, an increased number of genes coding for CAZymes classified to the module AA were identified in the genome of *A. parasiticus* compared to *A. flavus*. These enzymes are necessary for various functions, including the degradation of lignin and cellulose, the metabolism of metals, and the maintenance of iron homeostasis^34^. They can thus be seen as an indicator of the rather saprophytic lifestyle of this species in the soil habitat. A further indicator for the soil habitat is the fungal RiPP cluster, which was detected in the genome of *A. parasiticus* but not in the genomes of *A. flavus* and *A. minisclerotigenes*. Fungal RiPP clusters are associated, for example, with competitive defense and mycophagic activities^40^. Furthermore, various modifications, including two deletions and one insertion, were observed within the CPA gene cluster of *A. parasiticus*. Consequently, we did not detect any CPA in methanolic extracts of this species. As it can be assumed that multiple events caused these genetic variations, it can be suggested that there was no evolutionary pressure to preserve this cluster. Chalivendra et al.^41^ reported that *A. flavus* mutants, which were unable to produce CPA, exhibited lower virulence in causing ear rot disease. On the other hand, non-mutated strains that colonized maize were found to be strong CPA producers. The authors suggest that CPA is produced by fungi as a pathogenicity factor because of its cytotoxicity, which impairs the host’s defense response. Therefore, it can be inferred that the biosynthesis of CPA by *A. parasiticus* was not essential for this fungus, which is more saprophytic than parasitic. As a result, the maintenance of the CPA gene cluster was obviously discontinued. Alternatively, the inability to produce CPA may have shifted the fungus to the soil habitat. *A. minisclerotigenes* has already been isolated from soil as well as maize samples^12,13,42^, indicating its adaptation to both habitats. As for *A. parasiticus*, an increased number of enzymes necessary for lignin degradation compared to *A. flavus*, and additionally, magnesium and cobalt transport proteins encoded by species-specific genes of *A. minisclerotigenes*, might be indicators of adaptation for survival in the soil. In addition, a high CPA biosynthesis was observed for *A. minisclerotigenes* in this study. As mentioned previously, this could be advantageous for the infection of maize plants. Further, in comparison to the other species, *A. minisclerotigenes* seemed to be well-equipped for iron uptake. This was evident from the presence of a gene cluster coding for a siderophore, an iron-chelating molecule essential for the fungus’s high-affinity iron uptake^43^, in the genomes of both sequenced *A. minisclerotigenes* strains, but not in the genomes of the other species. A high bioavailability of iron, which is essential for the formation of co-factors for numerous enzymes and which is poorly available in most environments, could be a competitive advantage to this species^43^. *A. minisclerotigenes* appears to be particularly well adapted to survive in certain regions of Kenya^12,13^, where strains of this species were most likely responsible for outbreaks of acute aflatoxicosis^22^. *A. minisclerotigenes* MRI390 was shown to be genetically very closely related to strains that caused such outbreaks^28^. Due to the significant impact of the species *A. minisclerotigenes* on food safety, further research on it is recommended. The third analyzed species, *A. flavus*, is the most extensively studied species^7,44^, so the discussion will not focus on it.

In agreement with earlier investigations^31^, the comparison of the aflatoxin gene cluster generally showed a high degree of homology among the different species. However, relevant structural modifications were identified in all examined *Aspergillus* strains, and different corresponding chemotypes were observed. While the deletions within *afl*F and *afl*U in the genomes of *A. flavus* and *A. minisclerotigenes* have already been described^17,22,45^, the substitution detected within the *afl*L gene of *A. parasiticus* MRI410 has not been previously described, to the best of our knowledge. High levels of group 2 aflatoxins (AFB_2_, AFG_2_, AFM_2_) were measured in extracts of this strain, while there were hardly any group 1 aflatoxins (AFB_1_, AFG_1_, AFM_1_). This is highly atypical. Usually, the levels of group 1 aflatoxins are 10–100 times higher than those of group 2 aflatoxins^46–48^. The aflatoxin precursors detected in methanolic extracts of this strain, i.e., the detection of late precursors of group 2 but not of group 1 aflatoxins, aligns with this aflatoxin profile. The detected levels of AFB_1_ and AFG_1_ in methanolic extracts of *A. parasiticus*, which were found to be below the limit of quantitation, could potentially have been formed by other desaturases or through non-enzymatic processes in order to compensate for the one non-functional desaturation. It is highly likely that all subsequent steps in the pathway for the biosynthesis of group 1 aflatoxins are functional. Possible purposes for the production of aflatoxins for the fungus in general are discussed in the literature, such as their effect against insects or their role in protecting cells against the accumulation of reactive oxygen species^49,50^. However, the exact roles of the different aflatoxins, such as group 1 and 2 aflatoxins, as well as group B, G, and M aflatoxins, are not yet fully understood. Further research to understand why certain *Aspergillus* species produce G-group aflatoxins while others are also competitive with the deletion within *afl*F and *afl*U, or why *A. parasiticus* MRI410 is apparently able to survive without producing significant amounts of group 1 aflatoxins, while other *Aspergillus* strains primarily produce these compounds, would be interesting. The comparative study presented here focusing on three different toxicologically important *Aspergillus* species contributes to the ongoing research on aflatoxin and shows once again the complexity of the regulation and physiological background of mycotoxin biosynthesis by filamentous fungi.

## Materials and methods

### Fungal strains, genome sequencing and assembly, and functional annotation

The fungal strains *A. flavus* MRI19, originally isolated from tiger nuts grown in Spain^27^, *A. minisclerotigenes* strains MRI390 and MRI400, isolated from maize samples from Makueni, Kenya^28^, and *A. parasiticus* MRI410, isolated from a soil sample of a maize field in Katumani, Kenya^29^, were used for this study. The genomes of the strains were previously sequenced by us using short-read sequencing on a MiSeq instrument (Illumina, San Diego, USA) and additionally long-read sequencing on a PacBio Sequel instrument (Pacific Biosciences, Menlo Park, USA) for *A. flavus* MRI19 and *A. minisclerotigenes* MRI390 and MRI400. For *A. parasiticus* MRI410, long-read sequencing was performed on a Nanopore MinION Mk1C instrument (Oxford Nanopore Technologies, Oxford, UK) in addition to short-read sequencing. The software SPAdes (v. 3.14.1) was used with default software parameters to generate de novo hybrid assemblies of *A. flavus* MRI19 and *A. minisclerotigenes* MRI400, combining data from short and long-read sequencing, resulting in 68 and 52 contigs, respectively^27,28,51,52^. For the strains *A. minisclerotigenes* MRI390 and *A. parasiticus* MRI410, de novo assemblies of the long-reads were generated using the software Flye (v. 2.8.2) with default software parameters, resulting in 28 and 60 contigs, respectively^28,29,52,53^. Subsequently, the respective MiSeq data was aligned to the contigs using BWA (v. 0.7.17), SAMtools (v. 1.10), and QualiMap (v. 2.2.1), and the alignment was polished using Pilon (v. 1.23) and SAMtools (v. 1.10)^52,54–57^. Repeat masking was performed on the genome assemblies using RepeatMasker (v. 4.0.9) and Dfam (v. 3.0)^58,59^. Additionally, ab initio eukaryotic gene finding was conducted on both strands, allowing for the identification of partial genes using Augustus (v. 3.4.0), choosing *A. oryzae* as the closest species^60^. Identified genes were searched in the non-redundant protein sequence database performing a BLAST analysis (blastx-fast, *e*-value threshold 1.0*E-3). The respective taxonomic filter for each species was selected before mapping and annotating blast hits^61^. For the further functional annotation, InterProScan (v. 5.52-86.0) and EggNOG mapper (v. 2.1.0 with EggNOG v. 5.0.2) were used^62,63^. All steps were performed using OmicsBox (v. 17.0.2, BioBam Bioinformatics Solutions, Valencia, Spain), and default software parameters were used, unless otherwise specified. BUSCO (Benchmarking Universal Single-Copy Orthologs) analyses were performed using the lineage database ascomycota_odb10 to evaluate the completeness of the genome assembly data sets^64^.

### Whole-genome alignment

A whole-genome alignment was performed between the genome assemblies of *A. flavus* MRI19, *A. minisclerotigenes* MRI390 and MRI400, and *A. parasiticus* MRI410 using the Whole Genome Alignment Plugin of the CLC Genomics Workbench (v. 22.0.1, Qiagen, Hilden, Germany) without aligning based on individual chromosomes. The alignment had a minimum initial seed length of 15 nucleotides and a minimum alignment block length of 50 nucleotides. Based on this, the software calculated the average nucleotide identity using a minimum similarity fraction and a minimum length fraction of 0.8. The software further created a tree using the Neighbor-Joining algorithm on the distance matrix calculated from the seed matches between the individual genomes.

### Prediction of strain-specific genes and CAZymes

A BLAST database was generated of the nucleotide sequences of the predicted genes from the strains *A. flavus* MRI19, *A. minisclerotigenes* MRI390 and MRI400, and *A. parasiticus* MRI410 separately using OmicsBox (v. 17.0.2, BioBam Bioinformatics Solutions, Valencia, Spain). Then, the nucleotide sequences of the genes from each isolate were compared to those of the other three strains, respectively (tblastn, blast expectation value: 1.0*E-3). To predict specific genes of a strain, the results were filtered to include only genes that had either no or <50% similarity or <50% coverage to the nucleotide sequences of the other three strains. An enrichment analysis (Fisher’s exact test) was performed on the resulting genes against all genes of the respective strain (*p* value: 0.05)^65^. CAZymes were predicted in the gene sequences of the analyzed genomes using HMMER (*e*-value < 10^-15^, coverage > 0.35) against the dbCAN database and DIAMOND (*e*-value < 10^-102^) against the CAZy database using the dbCAN meta server (http://bcb.unl.edu/dbCAN2/blast.php)^32^. Genes identified by both tools were retained and categorized into the following modules based on their functionality: auxiliary activity (AA), carbohydrate-binding module, carbohydrate esterase, glycoside hydrolase (GH), glycosyl transferase, and polysaccharide lyase.

### Prediction of BGCs and comparison of the aflatoxin gene cluster

BGCs were predicted using antiSMASH (v. 6.1.0), employing the cluster finder algorithm for BGC border prediction and the relaxed detection strictness^66,67^. The aflatoxin gene cluster of the strains *A. flavus* MRI19, *A. minisclerotigenes* MRI390 and MRI400, and *A. parasiticus* MRI410, as well as that of *A. flavus* AF13^30^, *A. parasiticus* CBS 117618^31^, and *A. minisclerotigenes* CBS 117635^31^ was aligned using the CLC Genomics Workbench (v. 22.0.1, Qiagen, Hilden, Germany) to determine the average nucleotide identity, as described above.

### Quantitation of mycotoxins

For the quantitation of mycotoxins produced by *Aspergillus* sp. strains the following reference standards were used: aflatoxin B_1_ (AFB_1_, >99%), aflatoxin B_2_ (AFB_2_, >99%), aflatoxin G_1_ (AFG_1_, >99%), aflatoxin G_2_ (AFG_2_, >99%), and aflatoxin M_1_ (AFM_1_, >98%) all dissolved in acetonitrile (Sigma-Aldrich Chemie GmbH, Taufkirchen, Germany), aflatoxin M_2_ (AFM_2_) in acetonitrile (>98%), and aflatoxicol (AFL, >99%) (Cfm Oskar Tropitzsch GmbH, Marktredwitz, Germany), and cyclopiazonic acid (CPA) in acetonitrile (>99.9%) (Romer Labs Division Holding GmbH, Tulln, Austria). Spore suspensions containing 1.0 × 10^4^ spores per mL in Tween-80/NaCl-mixture (9 g/L NaCl [Carl Roth, Karlsruhe, Germany], 1 g/L Tween-80 [Serva, Heidelberg, Germany], 1 g/L agar [Agar-Agar Kobe Ι; Carl Roth, Karlsruhe, Germany]) were prepared of *A. flavus* MRI19, *A. minisclerotigenes* MRI390 and MRI400, and *A. parasiticus* MRI410. For spore counting, a Thoma cell counting chamber (Paul Marienfeld GmbH & Co. KG, Lauda-Königshofen, Germany) was used. MEA (15 g/L agar [Agar-Agar Kobe Ι], 30 g/L malt extract, 5 g/L tryptone/peptone ex casein [Carl Roth, Karlsruhe, Germany]), CYA (20 g/L agar, 30 g/L saccharose, 5 g/L yeast extract, 3 g/L sodium nitrate, 0.5 g/L magnesium sulfate heptahydrate, 0.01 g/L iron (II) sulfate heptahydrate, 1 g/L dipotassium hydrogen phosphate [Carl Roth, Karlsruhe, Germany], 0.5 g/L potassium chloride [Merck KGaA, Darmstadt, Germany])^68^, and YES (20 g/L agar, 150 g/L saccharose, 20 g/L yeast extract [Carl Roth, Karlsruhe, Germany])^69^ plates (Ø = 9 cm, 40 mL medium per Petri dish) were prepared. The plates were inoculated with 50 µL of each spore suspension as a one-point culture. Then, the fungal strains were cultivated for 7 days at 25 °C in the dark. At the end of the incubation period, two agar plugs (Ø = 8 mm) were taken from a colony, one from the center and one from the edge, using a sterile corer (*n* = 5 per strain and medium). The protocol for the following toxin extraction was based on the DIN EN ISO norm 16050:2011, with modifications as described^70^. One of the two agar plugs was transferred to a 2 mL tube filled with ceramic beads (Type A, Macherey-Nagel, Düren, Germany) and 1.25 mL of methanol:water (70:30, [v:v]) and 50 mg NaCl were added. The samples were homogenized (4 × 30 s, 6.0 m/s) using the FastPrep-24^TM^ (MP Biomedicals, Eschwege, Germany). Then, the extract was transferred into a new 2 mL tube. Subsequently, the second agar plug of the respective sample was transferred into the emptied 2 mL tube filled with ceramic beads and further 1.25 mL of methanol:water (70:30, [v:v]) and 50 mg NaCl were added. The homogenization procedure was repeated (4 × 30 s, 6.0 m/s) and the extract was transferred once again into a new 2 mL tube. To wash out the emptied 2 mL tube with the ceramic beads, further 1.25 mL of methanol:water (70:30, [v:v]) was pipetted into it and the tube was then processed for 30 s at 6.0 m/s in the FastPrep-24^TM^. The washing solution was transferred to a third 2 mL tube. The two extracts and the washing solution were centrifuged for 5 min at 16,200 × *g*. The supernatants from the three corresponding tubes were then combined und filtered using a 0.2 µm PTFE filter (Puradisc-13, Whatman^TM^, Merck, Darmstadt, Germany). The concentrations of the target analytes AFB_1_, AFB_2_, AFG_1_, AFG_2_, AFM_1_, AFM_2_, and AFL in the extracted samples were measured on a 1290 Infinity LC system (Agilent Technologies, Waldbronn, Germany) coupled with a Triple TOF 5600 mass spectrometer (AB Sciex, Darmstadt, Germany) using the method described in Schamann et al.^71^. Briefly, the separation was achieved on a Waters Cortecs UPLC C18 column (2.1 mm × 150 mm, 1.6 µm; Waters, Eschborn, Germany) equipped with a pre-column (Security Guard Ultra UHPLC C18; Phenomenex, Aschaffenburg, Germany). Gradient elution was conducted using aqueous ammonium acetate buffer (10 mmol/L) as eluent A and methanol as eluent B. The ion source of the MS was operated in positive ESI mode. For details, refer to Schamann et al.^71^. Each sample was measured in two different individual dilutions to ensure that the concentration of each analyte was within the calibrated concentration range (10–640 nmol/L). For data analysis, the software Sciex OS 2.0.1. (AB Sciex, Darmstadt, Germany) was used, and apart from this, it was performed as described in Schamann et al.^71^. Briefly, analytes were quantified based on standard curves applying linear regression with a weighting of 1/x^2^. The concentration of the lowest standard (0.01 µmol/L) was set as limit of quantitation of all the target analytes.

### Reporting summary

Further information on research design is available in the Nature Portfolio Reporting Summary linked to this article.

## Supplementary information

Description of Additional Supplementary Materials

Supplementary Data 1

Supplementary Data 2

Supplementary Data 3

Supplementary Data 4

Supplementary Data 5

Reporting Summary

## Data Availability

The genome assemblies of *A. flavus* MRI19, *A. minisclerotigenes* MRI390 and MRI400, and *A. parasiticus* MRI410 used in this study for comparative genomic analyses have been previously published^27–29^. Source data underlying Fig. 7 can be found in supplementary data sets 3/4 - currently states availability upon request, as well as the numerical values for the CAZymes screening. Parts of the data presented in this study are available only upon request from the corresponding author due to regulations of the Nagoya Protocol.
