# Supplementary material for: Comparative analysis of the genomes and aflatoxin production patterns of three species within the Aspergillus section Flavi reveals an undescribed chemotype and habitat-specific genetic traits
Source: Commun Biol. 2024 Sep 13;7:1134. doi: 10.1038/s42003-024-06738-w (PMC11399119; doi:10.1038/s42003-024-06738-w)
Supplement: Supplementary file 1 — Description of Additional Supplementary Materials [file 42003_2024_6738_MOESM1_ESM.pdf]

Description of Additional Supplementary Files

File name: Supplementary Data 1

Description: Enrichment analysis of strain-specific genes

File name: Supplementary Data 2

Description: : CAZymes in Aspergillus genomes

File name: Supplementary Data 3

Description: Aflatoxin levels in extracts of Aspergillus species

File name: Supplementary Data 4

Description: Analyte specific MS and MS/MS data of LC-MS analysis

File name: Supplementary Data 5

Description: The source data behind the graphs of figure

File name: Supplementary Data 6

Description: : Box plots visualization of the data in Figure 7.
