# Supplementary material for: Comparative analysis of the genomes and aflatoxin production patterns of three species within the Aspergillus section Flavi reveals an undescribed chemotype and habitat-specific genetic traits
Source: Commun Biol. 2024 Sep 13;7:1134. doi: 10.1038/s42003-024-06738-w (PMC11399119; doi:10.1038/s42003-024-06738-w)
Supplement: Supplementary file 4 — Supplementary Data 3 [file 42003_2024_6738_MOESM4_ESM.pdf]

Table S3. Aflatoxin levels (mean ± standard deviation) measured in extracts of *A. flavus* MRI19, *A. minisclerotigenes* MRI390 and MRI400, and *A. parasiticus* MRI410 after incubating the fungal strains for 7 days on MEA, CYA, and YES, respectively. Limit of quantitation (LOQ) of all analytes: 10 nmol/L.

Growth medium	Fungal strain	Aflatoxin B_1 [nM]	Aflatoxin B_2 [nM]	Aflatoxin G_1 [nM]	Aflatoxin G_2 [nM]	Aflatoxin M_1 [nM]	Aflatoxin M_2 [nM]	Aflatoxicol [nM]
**MEA**	*A. flavus* MRI19	35,723 ± 2,292	802 ± 72	< LOQ	< LOQ	228 ± 8	< LOQ	811 ± 176
	A. minisclerotigenes MRI390	26,192 ± 1,544	215 ± 19	< LOQ	< LOQ	166 ± 5	< LOQ	12 ± 2
	A. minisclerotigenes MRI400	20,228 ± 326	371 ± 12	< LOQ	< LOQ	117 ± 2	< LOQ	< LOQ
	A. parasiticus MRI410	< LOQ	5,572 ± 311	< LOQ	34,010 ± 1,363	< LOQ	98 ± 5	< LOQ
**CYA**	*A. flavus* MRI19	6,805 ± 475	77 ± 6	< LOQ	< LOQ	52 ± 2	< LOQ	316 ± 12
	A. minisclerotigenes MRI390	6,352 ± 365	37 ± 2	< LOQ	< LOQ	41 ± 2	< LOQ	< LOQ
	A. minisclerotigenes MRI400	21,577 ± 1,614	235 ± 20	< LOQ	< LOQ	177 ± 9	< LOQ	< LOQ
	A. parasiticus MRI410	< LOQ	1,059 ± 83	< LOQ	27,360 ± 1,298	< LOQ	44 ± 3	< LOQ
**YES**	*A. flavus* MRI19	28,651 ± 2,094	464 ± 34	< LOQ	< LOQ	447 ± 29	< LOQ	5,390 ± 485
	A. minisclerotigenes MRI390	59,687 ± 7,633	741 ± 124	< LOQ	< LOQ	747 ± 48	< LOQ	71 ± 6
	A. minisclerotigenes MRI400	155,617 ± 5,775	3,646 ± 217	< LOQ	< LOQ	1,726 ± 56	34 ± 1	464 ± 66
	A. parasiticus MRI410	< LOQ	15,700 ± 1,308	< LOQ	73,189 ± 4,267	< LOQ	347 ± 17	< LOQ
