# Supplementary material for: Comparative analysis of the genomes and aflatoxin production patterns of three species within the Aspergillus section Flavi reveals an undescribed chemotype and habitat-specific genetic traits
Source: Commun Biol. 2024 Sep 13;7:1134. doi: 10.1038/s42003-024-06738-w (PMC11399119; doi:10.1038/s42003-024-06738-w)
Supplement: Supplementary file 5 — Supplementary Data 4 [file 42003_2024_6738_MOESM5_ESM.pdf]

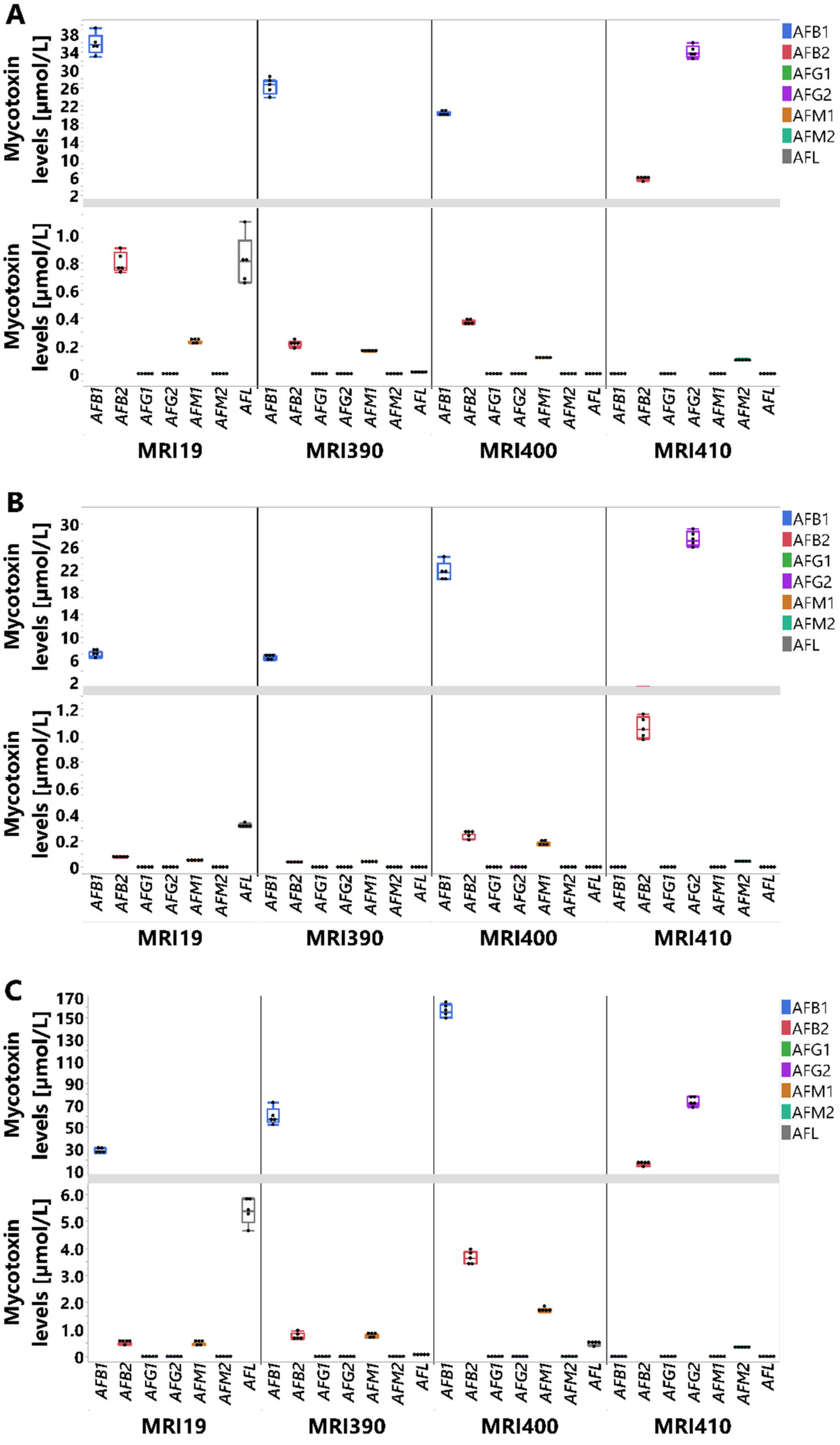

Supplemental Data 4, box plot visualization of Figure 7. Aflatoxin levels were measured by LC-MS in extracts of *A. flavus* MRI19, *A. minisclerotigenes* MRI390 and MRI400, and *A. parasiticus* MRI410 after 7 days of incubation on MEA (A), CYA (B), and YES (C), respectively. Limit of quantitation for all analytes: 0.01 µmol/L. AFB$_1$, aflatoxin B$_1$; AFB$_2$, aflatoxin B$_2$; AFG$_1$, aflatoxin G$_1$; AFG$_2$, aflatoxin G$_2$; AFM$_1$, aflatoxin M$_1$; AFM$_2$, aflatoxin M$_2$; AFL, aflatoxicol.
