# Supplementary material for: Comparative analysis of the genomes and aflatoxin production patterns of three species within the Aspergillus section Flavi reveals an undescribed chemotype and habitat-specific genetic traits
Source: Commun Biol. 2024 Sep 13;7:1134. doi: 10.1038/s42003-024-06738-w (PMC11399119; doi:10.1038/s42003-024-06738-w)
Supplement: Supplementary file 6 — Supplementary Data 5 [file 42003_2024_6738_MOESM6_ESM.pdf]

Supplementary Data 5. Analyte specific MS data of UHPLC-MS analysis in the positive ESI mode, showing the measured accurate mass, the mass deviation to the respective theoretical accurate mass, the molecular formula, and the retention time. Additionally, MS/MS data of the analytes are displayed (precursor ion and some major product ions).

**Analyte**	**Measured accurate mass [Da]**	**Deviation of measured mass [ppm]**	**Molecular formula**	**Retention time [min]**	**Precursor ion (MS/MS analysis) [m/z]**	**Selected product ions (MS/MS analysis) [m/z]**
**Versicolorin B**	341.0662	1.2	$C_{18}H_{12}O_7$	22.9	341.1	323.1, 313.1, 299.0, 285.0
**Dihydro-O-methylsterigmatocystin**	341.1020	1.0	$C_{19}H_{16}O_6$	19.7	341.1	326.1, 297.1, 285.1, 280.1
**Dihydro-11-hydroxy-O-methylsterigmatocystin**	357.0947	-1.6	$C_{19}H_{16}O_7$	13.0	357.1	311.1, 299.1, 285.1, 269.0
**Unknown metabolite**	329.0659	1.0	$C_{17}H_{12}O_7$ [a]	10.7	329.1	311.1, 301.1, 299.1, 273.0

[a] this molecular mass is only postulated
